# Decoding spatial attention with EEG and virtual acoustic space

**DOI:** 10.14814/phy2.13512

**Published:** 2017-11-28

**Authors:** Yue Dong, Kaan E. Raif, Sarah C. Determan, Yan Gai

**Affiliations:** ^1^ Biomedical Engineering Department Parks College of Engineering, Aviation and Technology Saint Louis University St Louis Missouri

**Keywords:** Auditory attention, brain– computer interface, EEG, phase, spatial attention

## Abstract

Decoding spatial attention based on brain signals has wide applications in brain–computer interface (BCI). Previous BCI systems mostly relied on visual patterns or auditory stimulation (e.g., loudspeakers) to evoke synchronous brain signals. There would be difficulties to cover a large range of spatial locations with such a stimulation protocol. The present study explored the possibility of using virtual acoustic space and a visual‐auditory matching paradigm to overcome this issue. The technique has the flexibility of generating sound stimulation from virtually any spatial location. Brain signals of eight human subjects were obtained with a 32‐channel Electroencephalogram (EEG). Two amplitude‐modulated noise or speech sentences carrying distinct spatial information were presented concurrently. Each sound source was tagged with a unique modulation phase so that the phase of the recorded EEG signals indicated the sound being attended to. The phase‐tagged sound was further filtered with head‐related transfer functions to create the sense of virtual space. Subjects were required to pay attention to the sound source that best matched the location of a visual target. For all the subjects, the phase of a single sound could be accurately reflected over the majority of electrodes based on EEG responses of 90 s or less. The electrodes providing significant decoding performance on auditory attention were fewer and may require longer EEG responses. The reliability and efficiency of decoding with a single electrode varied with subjects. Overall, the virtual acoustic space protocol has the potential of being used in practical BCI systems.

## Introduction

Although our human body frequently receives mixed sensory inputs from various spatial locations, the brain is able to focus on a particular input source or point in space. Selective attention can significantly improve perceived signal‐to‐noise ratios by suppressing undesired sensory inputs from other sources, such as in the “cocktail‐party scenario” (Saberi et al. 1991; Arbogast et al. 2005). Decoding spatial attention based on brain signals can be useful in brain–computer interface (BCI) for paralyzed patients, such as brain‐controlled wheelchairs. Once the user's destination in terms of a horizontal angle is decoded, the wheelchair can then move in the precise direction.

Both Electrocorticography (ECoG) (Dijkstra et al. 2015) and Electroencephalography (EEG; Power et al. 2012; Lauteslager et al. 2014; O'Sullivan et al. 2015) are known to be capable of providing information for decoding auditory attention to multi‐talker speech. For patients without cortical implantation, EEG is preferred as it provides a non‐invasive and portable approach for monitoring brain signals. One type of EEG signals (i.e., asynchronous signals) is voluntarily initiated by the brain in the absence of sensory stimulation. The application typically requires substantial training of the human subjects. The second type of EEG is called synchronous signals, which depend on external light or sound stimulation and require little training (Rupp 2014). Previous studies have attempted to develop brain‐controlled wheelchairs based on both types of signals (Cheng et al. 2002; Bastos et al. 2011; Chai et al. 2014; Mohebbi et al. 2015). Operations of those wheelchairs often involve certain *mapping* between the sensory stimulation (e.g., light or sound) and the patient's intention (e.g., action or destination). For example, in Mohebbi et al. (2015), wheelchair‐movement commands, such as “Left” and “Right”, were represented by a set of visual patterns on a screen. When the subject looked at a pattern, evoked brain signals were classified and used to drive the wheelchair. This type of protocol does not decode spatial attention and differs substantially from human's natural body movements.

A more intuitive and accurate approach is to directly extract the user's spatial attention in terms of a horizontal angle (in the case of wheelchairs, the vertical angle is irrelevant). Visual spatial attention based on EEG has been extensively studied (e.g., Kelly et al. 2005; Rihs et al. 2007; Yamagishi et al. 2008; Trachel et al. 2015; Samaha et al. 2016). There are fewer studies on auditory spatial attention, which often involve free‐field sound presented by two or more loudspeakers placed at different spatial locations (Schreuder et al. 2010; Kim et al. 2011; Thorpe et al. 2012). Whether using visual or auditory stimulation, a large number of speaker positions or light sources need to be physically displayed around the user to achieve a high spatial resolution.

**Figure 1:**
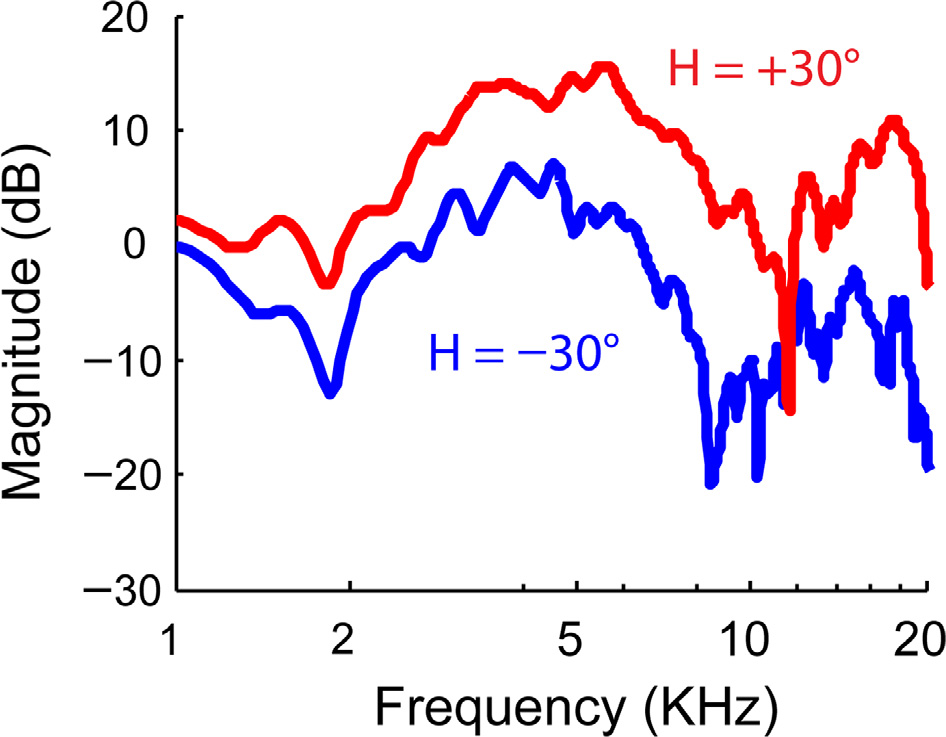
HRTFs recorded in the right ear of a subject to two speakers placed in the horizontal plane with two different angles, −30° (left to the subject) and +30° (Algazi et al. 2001).

The present study explored the possibility of using virtual acoustic space as a stimulation protocol for decoding spatial attention. The creation of virtual acoustic space was based on head‐related transfer functions (HRTFs). Normally, the human head and pinnae add time and frequency properties to a free‐field sound before it reaches the eardrum (Gardner and Gardner 1973). Those spectral and temporal properties carry information of the source location. Figure 1 shows two examples of HRTFs from Algazi et al. (2001). Those functions were derived from acoustic recordings in humans using in‐ear microphones, with loudspeakers placed at various spatial locations. Each pair of HRTFs (left and right) is essentially two filters that are unique for sound coming from a spatial location. The spatial location can be represented by a horizontal angle, a vertical angle, and a distance. In contrast, when sound is presented via headphone, it is perceived as coming from inside the head due to the lack of spectral filtering (Plenge 1974). The missing spatial information can be restored by artificially filtering the sound, using HRTFs, a procedure called *externalization* (Hartmann and Wittenberg 1996). Theoretically, the virtual acoustic space technique allows creating sound that carries spatial features for any virtual location. Compared with the study by Lopez‐Gordo et al. (2015) that generated monaural sound to either the left or right ear, our configuration allowed the presentation of sound coming from any virtual location, such as 30° and 80° to the right.

After the creation of spatial sound, the next step is to decode auditory attention, that is, how can a listener's attention to one of multiple sounds be accurately decoded based on EEG signals? The decoding algorithm at this step does not necessarily relate to spatial attention. If the sound token being attended to can be inferred from EEG signals, its corresponding spatial location will indicate the listener's spatial attention. One method for presenting sound is the oddball paradigm, in which sound tokens are presented in sequence (i.e., one at a time). Features of the event‐related potentials to a rare auditory target, such as the P300, are used as the decoding metrics (Schreuder et al. 2010).

Alternatively, a forced‐attention paradigm (Berlin et al. 1973) requires that the listener pays attention to one of the streams presented concurrently, while ignoring the others. To successfully infer which sound was attended to by the listener, a unique stimulus feature is tagged to each sound. For example, in the study by Kim et al. (2011), sound coming from the left speaker had a modulation frequency of 37 Hz, whereas sound from the right speaker had a modulation frequency of 43 Hz. Later, a “phase‐tagging” approach was developed (Lopez‐Gordo and Pelayo 2013; Lopez‐Gordo et al. 2015), in which the modulation frequencies for up to six concurrent speech sounds were the same, while each sound had a unique phase value.

Here, we combined the phase‐tagging decoding algorithm with virtual acoustic space as the experimental paradigm. A focus of this study was to test the feasibility of the approach in BCI applications related to spatial attention, particularly when the spatial information is presented virtually. One critical aspect is the reliability of the decoding performance over multiple sessions and days. Therefore, we recorded a large number of trials (800 trials per condition) for each subject. Another aspect is the efficiency of the decoding, mostly determined by the duration of EEG response required to form a reliable decision variable. We found that the decoding performance of a single electrode can be highly reliable for a small number of subjects using EEG responses of 90 sec or less. For the majority of subjects, longer responses would be required to achieve a stable performance that is safe to be used in practical BCIs. In addition, our study explored a visual‐auditory matching paradigm that closely resembles BCI applications involving spatial attention, such as a wheelchair.

## Methods

### General setup

Eight human listeners (aged 19−40; four females) participated in the study. All listeners passed a screening for normal hearing, judged by pure tone thresholds ≤ 20 dB HL for frequencies of 0.25, 0.5, 1, 2, and 4 kHz. The experimental protocol was approved by the Institutional Review Board of Saint Louis University.

**Figure 2:**
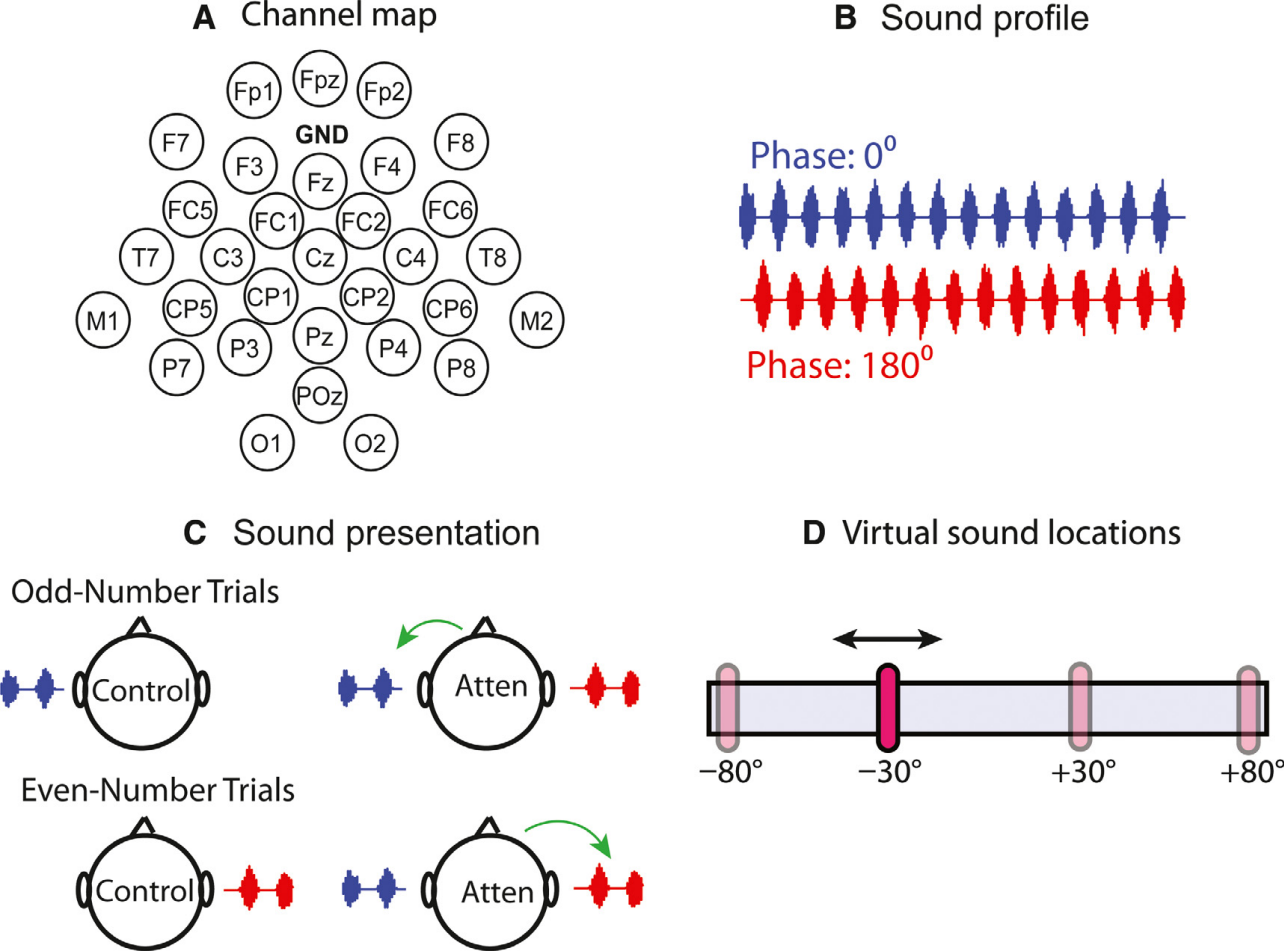
Experimental configurations. A, the electrode‐channel map for the 32‐channel EEG system. B, amplitude‐modulated noise. The sound corresponding to the left side (blue) had a 0° phase in the amplitude modulation. The sound corresponding to the right side (red) had a 180° phase. C, Experiment I‐a. Each sound was presented to one ear. Only one sound was presented in the control trials (*left* column); the side alternated between odd and even numbered trials. The two sounds were presented simultaneously in the attention trials (*right* column); the green arrow indicates the required attention that alternated between odd and even numbered trials. D, virtual horizontal locations (−80°, −30°, +30°, and +80°) for Experiments I‐b and II. In I‐b, each sound was presented to both ears with a virtual location, −80° or +80°; the virtual location or attention alternated the same way as I‐a. In Experiment II, a slidebar occurred at one of the two locations randomly. The subject was required to pay attention to the sound that had a virtual location matching the location of the slidebar. Two of the four locations were selected for each subject (Table 1).

Experiments were conducted in a double‐walled sound booth (8′ x 8′ x 8.1′; Noise Barriers LLC, Illinois). EEG signals were measured with a 32‐channel portable system (eego‐sports; ANT Neuro) that comprised a head cap, an amplifier, and a Windows tablet computer. The sampling frequency was 500 Hz. Figure 2A shows the electrode arrangement. The ground electrode was located in between Fpz and Fz. Only 31 electrodes were active since the additional one (CPz; not shown) served as the reference electrode. Sound was generated with a sampling frequency of 44.1 kHz using MATLAB (MathWorks) and delivered to a StimTracker (Cedrus). The StimTracker is a device that relays the sound to a headphone (Sennheiser HD 280 PRO), while sending a precisely timed trigger signal to the EEG amplifier at the sound onset. We measured the time jitter of the StimTracker, which was smaller than the time resolution of the EEG signals (2 msec).

Noise and speech carriers were amplitude modulated at 5 or 7 Hz. According to Lopez‐Gordo and Pelayo (2013), a 5‐Hz modulation creates a half‐period of 100 msec that approximately matches the N1‐P2 complex in an ERP. Meanwhile, 2 to 8 Hz is the range of frequencies for which speech‐envelope information is linearly related to EEG (Pasley et al. 2012; O'Sullivan et al. 2015). Because EEG responses are low frequency in nature, they can only reflect slow variations of sound amplitude, not the rapid carrier. Here, we used 5 Hz to modulate speech sound. We also used 7 Hz to modulate the noise to explore whether a slightly higher frequency can work with similar efficiency. Higher frequencies have the advantage of providing more modulation cycles in a fixed time duration, and thus more phase measurements. On the other hand, when the frequency is too high, phase differences measured with absolute time differences will be too small to be reliably detected. For example, when there are two competing sound sources, the time delay associated with the phase delay between the two sounds for a 7‐Hz modulation is 1000/7/2 = 71.4 msec. As the frequency increases, the time difference further decreases, which will require higher accuracy of the system for reliable phase measurements.

Human HRTFs were obtained from Algazi et al. (2001). The distance between the speakers and the human subject was always 1 m in that study. We randomly selected a recording subject from their study and used the HRTF filters obtained from this subject for all of our experimental conditions. The vertical angle was fixed at 0° while the horizontal angle varied. HRTF filtering was applied to the sound after amplitude modulation and phase tagging. The sound level was approximately 50−55 dB SPL measured by a sound level meter (Extech, MA) in the monaural condition. The exact level of binaural sound varied over a larger amount determined by the HRTF filters, which contained the interaural level cues.

### Experimental paradigms

Table 1 summarizes the experimental conditions. Briefly, Experiment I involved sound presentation or spatial attention alternating between the left and right ear/side of the subject on odd and even numbered trials. The goal of Experiment I was to replicate previous monaural conditions in a left/right manner (Lopez‐Gordo et al. 2015) as a preparation for exploring the virtual‐space paradigm. In experiment II, spatial attention was guided by the location of a visual target that randomly appeared among two locations on a computer screen. For each subject, it took multiple days to finish all the recording sessions.

**Table 1:** Experimental conditions and sound profiles. Note that not all subjects participated in all tasks

Experiment	Tasks	Sound	Positions	Subjects
I	Alternating attention	I‐a: noise, no‐HRTF	Left ear vs. right ear	S1, S2, S3, S7, S8
		I‐b: noise, HRTF	(−80°, +80°)	S3, S4
II	Localization	II: Speech, HRTF	(−80°, +80°),	S1, S5
			(−30°, +30°),	S2, S6
			(+30°, +80°)	S3, S4, S7

*f*_mod_ = 7 Hz for noise, and *f*_mod_ = 5 Hz for speech. HRTF, head‐related transfer function.

#### Experiment I: Alternating attention

The first experiment used an alternating paradigm with auditory cues to guide spatial attention. The sound was amplitude‐modulated random noise (4‐s long) with *f*_*mod*_ = 7 Hz. Here, the envelope was a “transposed tone” (Fig. 2B) rather than a sinusoid because the transposed envelope elicits better synchronization in the cortex (Hartley and Isaiah 2014). In Experiment I‐a, each sound was monaural without HRTF implementation. Since the phases of the two sounds differed by 180°, the peaks in the first sound coincided with silent periods in the second sound, and vice versa (Fig. 2B).

Figure 2C shows the experimental condition for I‐a. In the **control** (*left* column), one sound (0° phase; blue) was presented to the left ear on odd‐numbered trials, and the other sound (180° phase; red) was presented to the right ear on even‐numbered trials. The frequency spectra of the noise also differed (left, 2−8 kHz; right, 5−15 kHz) so that the subject could distinguish the two sounds (Kim et al. 2011). The subject was required to be marked on an answer sheet whether the sound was presented to their left or right ear, which ensured that they were attentive to the sound. In the **attention** condition (Fig. 2C, *right* column), a 0.5‐sec cue signal was presented to one ear, followed by both sounds presented concurrently for 4 sec, one for each ear. During the 4 sec, the subject was required to pay attention to the cued ear and ignore the sound in the other ear. The cue was a shortened version of the target sound and alternated between ears on odd‐ and even‐numbered trials. For both control and attention conditions, a total of 100 trials (50 for each side) were collected during each recording session with an inter‐sound interval of 4 sec. For each subject, three sessions were obtained for the control condition. Thus, the total trial number, *N*, was 300. Eight sessions were obtained for the attention condition (*N* = 800). An attention session typically lasted for 14 min.

Experiment I‐b was similar to I‐a, except that each sound was binaural. That is, the sound source or attention still alternated between left and right on odd and even numbered trials; a cue preceded the attended sound. The difference was that HRTF filtering was performed to alter the virtual location of the sound between left and right (−80° or +80°). This condition served as a transition to Experiment II. Here, we explored the possibility of using HRTF to shift the virtual location of the sound.

#### Experiment II: Localization

In Experiment II, we explored the possibility of matching auditory attention to the location of a visual target on a computer, which we call a visual‐auditory matching paradigm. Two amplitude‐modulated sentences (Calandruccio and Smiljanic 2012), one male and one female, were used for all the subjects. *f*_mod_ = 5 Hz. The sentences were filtered by HRTFs after amplitude modulation, each fixed for a virtual location. Figure 2D shows possible virtual locations for the HRTFs; three pairs of locations were assigned to the subjects (Table 1). At the beginning of each trial, the two sentences were presented alone in sequence, each given a time slot of 2 sec (the sentence durations were shorter than 2 sec). Corresponding EEG signals were extracted as the control response. Afterward, a slidebar appeared randomly at one of the two locations (Fig. 2D) on a computer. Note that the subjects were presented with three different sets of locations (Table 1) to test the idea that the virtual‐space paradigm has the flexibility of presenting sound from any location.

Meanwhile, the two sentences were presented concurrently for 4 sec, and the subject was required to pay attention to the sentence having a virtual location that best matched the location of the slidebar. The gaze of the subject was not fixed because the subject needed to track the location of the slidebar. A recording session typically lasted for 20 min with a total of 80 trials obtained. (Collecting 100 trials would have been too tedious for the subjects.) Ten sessions were performed for each subject so that N was still 800 to match Experiment I.

Note that since we did not measure individualized HRTFs or simulate room reverberations, the speech did not sound fully “externalized” with the desired distance (1 m) in front of the subject. Nevertheless, the subjects reported that they were able to spatially separate the sound sources and mentally map the perceived sound locations to the locations of the visual targets. A brief psychophysical study was performed in the end to verify the behavior (see Behavioral verification).

### EEG signal processing and classification

We used an independent component analysis (ICA) approach (Zhou and Gotman 2009) to remove EEG artifacts. This algorithm worked effectively most of the time for the subjects reported here. Initially there was one additional subject whose artifacts could not be removed by this algorithm; the subject was excluded from the study. Signals were then bandpass filtered between 0.1 and 30 Hz, using a 50th‐order FIR filter. On each trial, a 4.5‐sec response was extracted after the sound onset because we found that the half second after the stimulus offset also contained useful phase information. A constellation approach similar to the method presented by Lopez‐Gordo and Pelayo (2013) was implemented for signal classification. Briefly, EEG signals during sound presentation were extracted and the root mean square of the signal was normalized to 1. This normalization was necessary because we combined training trials from multiple days and the electrode impedances may vary from day to day. Next, Fourier transform was performed to the normalized signal with a frequency resolution of 0.1 Hz. The frequency component at *f*_*mod*_ (5 or 7 Hz) was projected to the complex plane with the *x*‐axis being the real and the *y*‐axis being the imaginary components. Symbols were classified based on their locations and proximity in this complex plane.

**Figure 3:**
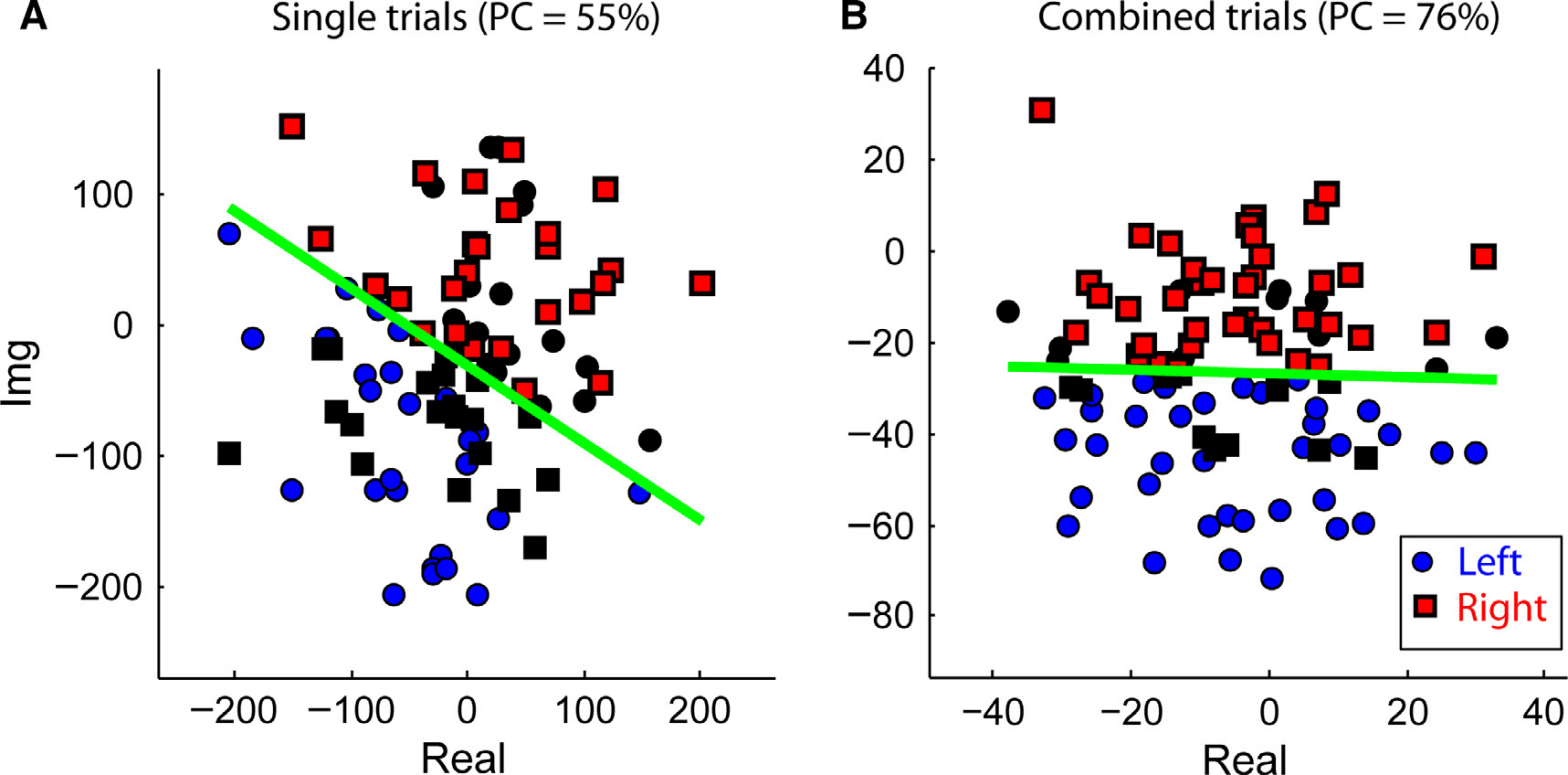
An example of the attention response (e.g., the EEG frequency component at 7 Hz) obtained from Subject S2, Electrode Channel Cz, during Experiment I‐a. A, linear classification result based on single trials (50 single trials for each side). Each point corresponds to a 4‐sec sound presentation. The trials were obtained from one recording session. B, linear classification result based on combined trials across eight sessions (50 combined trials for each side). Each point was an average of 20 trials. Circles represent EEG signals during left‐side attention; squares represent signals during right‐side attention. Colorful symbols are correct classifications; black symbols are incorrect classifications. PC, percent correct rate.

Figure 3A shows an example of single‐trial responses obtained in Experiment I‐a with Electrode Channel Cz. Circles and squares represent responses to the binaural sound when attention was directed to the left and right sides, respectively. A linear discriminator based on the least squares approach was then applied to classify the responses (Semmlow and Griffel 2014). When a linear boundary was drawn to separate the symbols with a threshold value of 0.5, only 55% of the trials were correctly classified (symbols with colors) based on single trials.

Because some previous auditory attention studies used up to 20 sec of duration (Kim et al. 2011; Lopez‐Gordo et al. 2015) on each trial, we first tried combining five trials and used the mean vector as a decision variable. Except for two subjects, this combination was insufficient for producing relatively consistent performance over different recording sessions. We found that at least 90 sec of responses, obtained by combining 20 trials, were needed to form a reliable decision variable for most of the subjects. In addition, it was insufficient to train the classifier using responses obtained from only one session. Instead, when one of the *m* sessions obtained under the same condition was used as a testing session, the other *m*–1 sessions were combined to form the training pool. Specifically, each time 20 trials in the same class obtained in a testing session were randomly selected to form a test point. Meanwhile, 200 training points were formed for each class, with every training point being a combination of 20 trials that were randomly selected from the rest of the pool of the same session, as well as the other *m*–1 sessions. This approach was essentially the same as the commonly used “leave‐one‐out cross‐validation” approach except that we combined trials. The linear discriminator described above was then applied. The process was repeated 50 times for each class. This way, we obtained a session‐by‐session performance so that we could examine the consistency of the decoding.

For BCI applications, at least one fixed electrode channel that performs consistently over time will be needed to make a decision. The identification of the “best channel” was based on examining the phase information contained in all of the *m* recorded sessions. With 800 trials (400 for each class) in total, the average of half of the trials in each class (i.e., 200) was used as a testing point; the other half served as the training point. The vector distances between the test point and each of the two training points were measured. The test point was assigned to the class of the training point with which it had the shorter vector distance. A total of 400 repetitions were performed. A correct rate can be computed by dividing the number of correct decisions by the number of repetitions. The channel that had the highest correct rate was chosen as the “best channel”. The performance of this channel over multiple sessions was used as a measure for “consistency” required by practical BCI systems.

## Results

### Single‐sound location can be decoded from most electrodes

**Figure 4:**
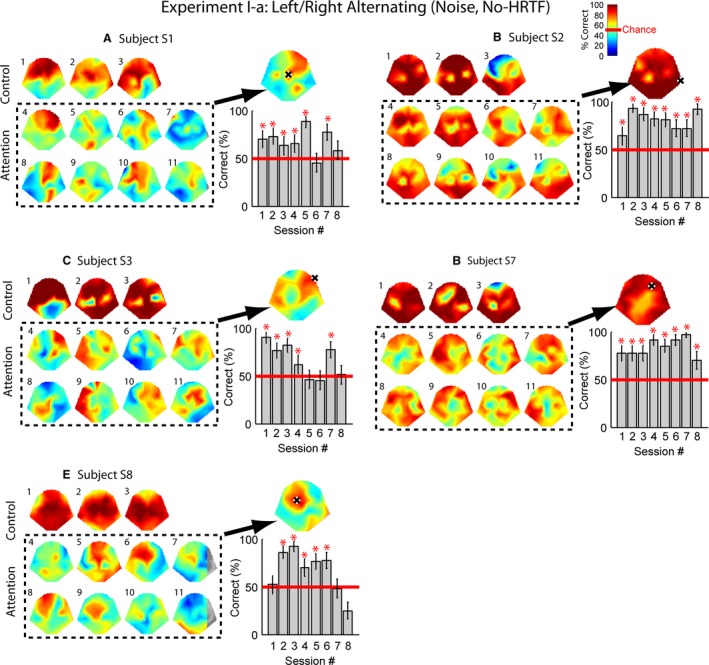
Decoding performance for Experiment I‐a. Each sound was a monaural amplitude‐modulated (*f*_mod_ = 7 Hz) noise without HRTF filtering. Control: a single sound at either ear was presented. Attn: both sounds were presented simultaneously, and the subject was required to alternate attention on odd and even numbered trials. The decoding variable was computed based on 90‐sec EEG responses through a combination of 20 trials, each consisting of a 4.5‐sec EEG signal. In each plot for a particular subject, the small heat maps indicate the correct rates for control (top) and attention (dashed rectangle) conditions obtained from 100 trials. The number next to each heat map marks the session number. 100 iterations were performed for every session by randomly selecting 20 trials each time. The color ranging from blue to dark red represented 0 to 100% correct rates. The large heat map on the upper right corner of each plot shows the phase sensitivity combining all 800 trials, using a correlation approach. The black‐and‐white marker indicates the electrode selected according to the maximum performance. The bar plot on the lower right is the session‐by‐session performance of this selected electrode. The red lines mark the chance performance (50%). Error bars indicate 95% confidence intervals. * Significantly above chance (*P* < 0.05).

**Figure 5:**
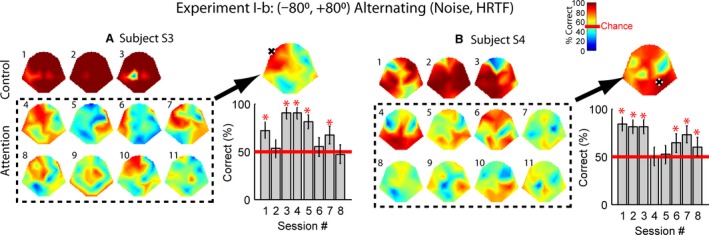
Decoding performance for Experiment I‐b in the same format of Fig. 4. Each sound was a binaural amplitude‐modulated (*f*_mod_ = 7 Hz) noise with HRTFs representing horizontal angles of −80° or +80°. Control: a single sound at either location was presented. Attn: both sounds were presented simultaneously, and the subject was required to alternate attention on odd and even numbered trials.

**Figure 6:**
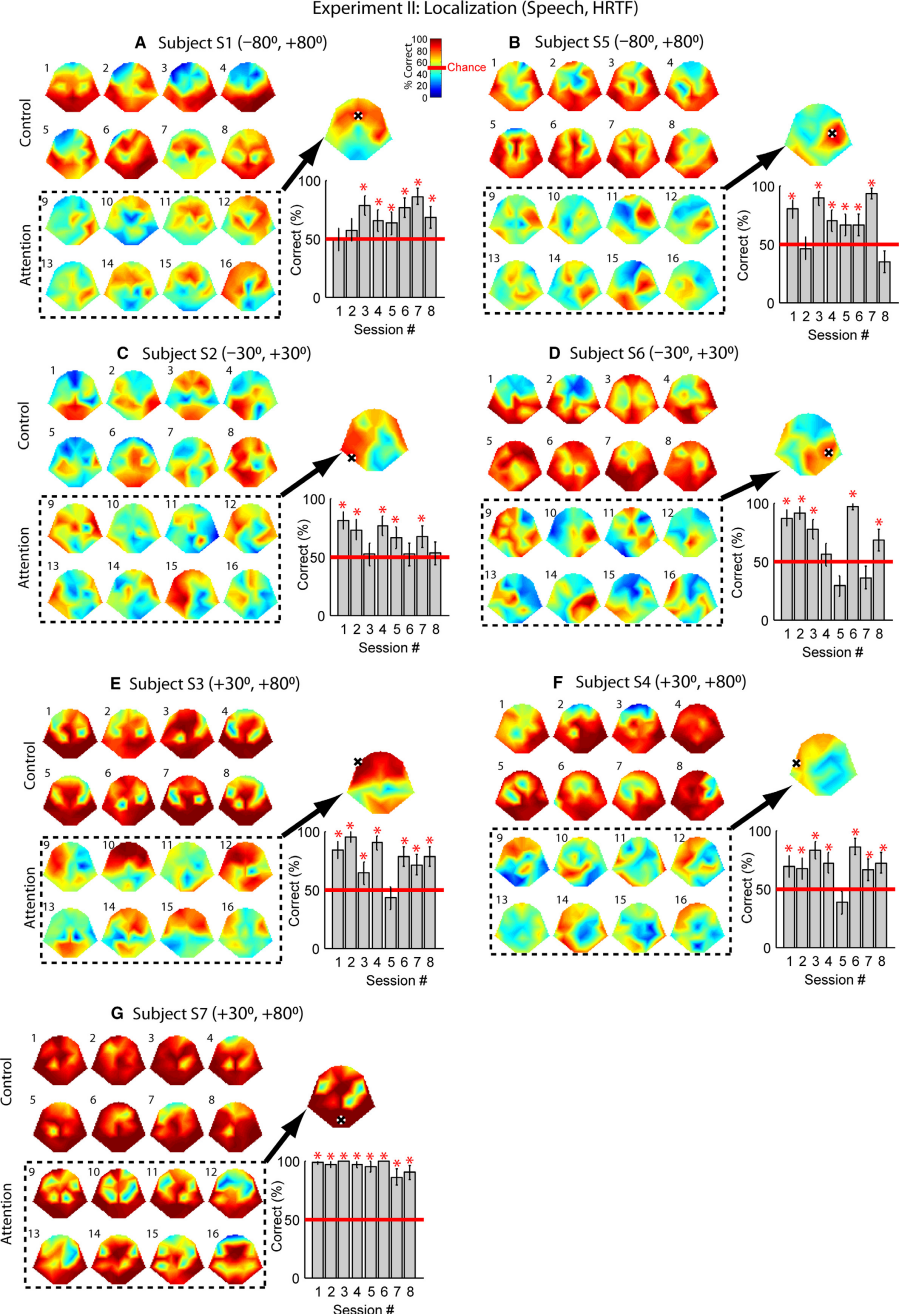
Percent Corrects of Experiment II for all subjects in the same format of Figure 4. Two speech sentences were amplitude modulated with *f*_mod_ = 5 Hz and filtered with HRTFs representing two horizontal angles specified in the title of each plot. Control: a single speech sound at one of the two locations was presented. Attn: both sounds were presented simultaneously, and the subject was required to pay attention to the sound matching the location of a visual target on a computer.

Overall, when a single sound was presented, whether it was monaural (Fig. 4) or binaural (Figs. 5 and 6), the sound phase and thus the virtual location can be decoded with high accuracy over most of the electrodes.

The top row of the small heat maps in each panel of Figure 4 shows the control performance of Experiment I‐a, when a single monaural amplitude‐modulated noise was presented to alternating ears (i.e., control). Each session contained 100 trials. Green in the heat map indicates chance performance, and warm colors represent high correct rates. Although the patterns may vary, all subjects had near 100% correct rates with some of the electrodes. A typical pattern was observed with Subjects S2 and S7 (Fig. 4, B and C). Small areas near C3 and C4 (i.e., the primary motor cortex) tended to have lower performance (*χ* ^2^ test, *P* < 0.05), whereas most other electrodes were found to be highly sensitive to single‐sound modulation phases. This pattern would reappear in later conditions. Occasionally the frontal or occipital electrodes exhibited low correct rates (*χ* ^2^ test, *P* < 0.05).

In Experiment I‐b, monaural sounds were replaced with binaural sounds, and HRTFs were applied to alternate the virtual sound sources between the left and right ears. The sound was the same amplitude‐modulated noise with phase tagging as in I‐a before going through the HRTF filters. In the control condition, most electrodes of the two subjects were able to encode the sound phases (Fig. 5, top rows), and thus the location (−80°, +80°). Subject S3's control patterns were similar to her patterns in Experiment I‐a in that the lowest performance was observed around C3 and C4. Subject S4 showed the lowest performance at the frontal and/or occipital electrodes (*χ* ^2^ test, *P* < 0.05).

When the visual‐auditory matching paradigm was used to guide the attention, control responses were recorded while the subject listened to each individual sentence played in sequence before the matching occurred. The top two rows in Figure 6 are control performance. For most of the subjects, the performance was similar to Experiment I, that is, the majority of electrode channels can reliably encode the sound phase with the lowest performance occurring around C3, C4, or the frontal electrodes (*χ* ^2^ test, *P* < 0.05). Subject S2 (Fig. 6C) had fewer electrodes that could decode the single‐sound phase compared with the other subjects.

### Auditory attention can be reliably detected from limited number of electrodes

When two concurrent sounds with unique phases and spatial features were presented to the listeners, covert attention to a single source may be correctly detected after averaging over 20 trials with different levels of reliability across subjects. The electrodes that reflected forced attention were generally fewer than the electrodes that reflected the modulation phase of single sounds (i.e., control).

Figure 4 (dashed rectangles) shows all the attention sessions obtained during Experiment I‐a with monaural sounds. In each session, the decision variable was derived from an averaged response over 20 individual trials, which made a total of 90‐sec response. For Subjects S2 and S7 (Fig. 4, B and D, dashed rectangles), the majority of electrodes could reliably reflect the attended sound phase, and thus the virtual location of the visual target. The best electrodes stayed relatively consistent over sessions. For the other three subjects (Fig. 4, A, C, and E, dashed rectangles), the electrodes that could reliably decode the sound phase varied across sessions and were considerably fewer than electrodes that reflected the phase of a single sound.

For the purpose of BCI applications, an electrode channel has to be selected for decision making. Given the variation of the brain patterns, one may wonder how practical it is to use the phase‐tagging approach when the electrode position is fixed. To locate a good‐performing electrode for each subject, a correlation approach was used to examine the phase sensitivity by combining all 800 trials (see Methods; Fig. 4, large *upper‐right* heat map in each panel). Although brain patterns obtained with the individual sessions varied, the phase sensitivity shown by the combined plots indicated salient decoding result that did not get washed out by averaging over hundreds of trials.

Next, the electrode that showed the maximum sensitivity was chosen (black and white marker). The bar plot underneath the large heat map shows the correct detection rates of this single electrode over multiple recording sessions. The red line marks chance performance at 50%. The error bars indicate the 95% confidence intervals for individual channels, plotted in a way similar to Lopez‐Gordo et al. (2015). Here, the confidence interval was computed as $\pm 1.960(\sqrt{\frac{\hat{p}\left(1-\hat{p}\right)}{n}})$, where $\hat{p}$ was the percentage of correct decoding and n was the number of samples (Lock et al. 2017). Red asterisks indicate significant performance above chance (*P* < 0.05). Out of the eight sessions, Subjects S1, S3, and S8 showed five or six significant sessions, whereas Subjects S2 and S7 showed significant performance for all eight sessions. The average performance for the sessions above chance ranged between 71% and 90%.

In Experiment I‐b, binaural sounds were presented with virtual locations determined by the HRTF filters. The horizontal locations for the two sounds were −80° and +80°. Similar to Experiment I‐a, five or six sessions out of eight were above chance with mean values of 83% and 78% for the two subjects.

In Experiment II, spatial attention was guided by a visual object that randomly appeared in one of the two locations (Fig. 2D), corresponding to two virtual locations of the sounds. With each pair of locations, two or three subjects participated in the test. The subject was required to pay attention to the sound location that best matched the location of the visual target. Subjects S1 and S5 had the largest angular separation, (−80°, +80°), symmetrical to the front center. A smaller angular separation was used for Subjects S2 and S6, (−30°, +30°). For Subject S3, S4, and S7, both sound sources, as well as the visual targets were located off center to the right (+30°, +80°).

Subject S7 was the only one whose majority of electrodes can accurately encode auditory attention (Fig. 6G, *left*). The performance was almost as good as a typical control performance. The decoding accuracy based on Electrode POz remained near 100% for all recording sessions (Fig. 6G, *lower right*). For the rest of the subjects, the electrodes that encoded attention were fewer and variable. When a good performance electrode was chosen, 4 to 7 out of 8 sessions showed significant accuracies. For those subjects, the average performance of the above‐chance sessions ranged between 73% and 84%. We did not observe an effect of the exact two locations of the sound pairs on decoding accuracy.

### Behavioral verification

Since variations of decoding performance were observed across the subjects, there was a likelihood that the variations were caused by the subject's ability to behaviorally map the visual target with the virtual space sound. Therefore, we added a brief psychophysical test with three of the subjects (S2, S3, and S7) without EEG recording. In each trial, the subject was presented with all four possible locations (−80°, −30°, +30°, and +80°). The subject was asked to select from a list of four sentences (presented in a random order) that matched the position of the virtual target (i.e., the slidebar). A total of 20 trials were collected for each subject. The correct identification rate of the subjects ranged from 95 to 100%. Therefore, the decoding performance was unlikely to be caused by inaccurate mapping between the virtual acoustic space and the visual target.

## Discussion

In this study, we explored the possibility of using virtual acoustic space as a stimulation protocol for constructing BCI that involves the decoding of spatial attention. There were two major components involved in the sound presentation. One was to create the sense of spatial properties for headphone‐delivered sound. The second was to tag each sound source with a unique modulation phase. In theory, the two components should work independently. Note that the inclusion of HRTFS was not to make the decoding easier but to make a better BCI practice.

### Virtual acoustic space is an effective stimulation approach for measuring spatial attention

There can be several advantages of using the virtual acoustic space when being applied to BCI. (1) Portable. When using light sources or loudspeakers, traditional BCI practices involving spatial attention will require that multiple visual or auditory sources are physically placed around the user and the position of each source has to be adjustable for a fine spatial resolution. In contrast, sound stimulation based on virtual acoustic space can be generated by a headphone, which is easier to carry. (2) High spatial resolution. Sound can be created from any virtual spatial angle. Even for angles that were not specifically recorded in the HRTF recordings, new filters can be interpolated based on recordings for other locations using a method developed by Zotkin et al. (2004). (3) Vision free. The user's gaze can be engaged in other activities.

In the present study, the smallest angular separation was 50° (Fig. 6, E and F). In theory, as long as the subject can mentally separate the sound sources, the detection accuracy will mostly depend on the efficiency of the auditory‐decoding algorithm (e.g., phase tagging). For example, if the subject could reliably separate two sources that were 10° apart, the detection performance should be similar to the 50°‐separation performance.

In Experiment II, the subject was presented simultaneously with two sounds having different virtual locations. Meanwhile, a visual target (i.e., a slidebar) appeared on the screen, and the subject attended to the sound that best matched the location of the target. This visual‐auditory matching paradigm ultimately resembled applications of auditory‐spatial decoding in real world. For example, in the application of a brain‐controlled wheelchair, the user will first have a destination in mind. Next, the user will pay attention to the sound source that best matches the destination. In other words, the physical environment will be aligned with the virtual acoustic environment. By decoding the attended sound source, spatial attention is inferred. The complexity of our experimental paradigm was higher than what have been used in previous studies. For example, in Lopez‐Gordo et al. (2015), sentence onsets were staggered with a 0.5 or 0.7 sec delay. Subjects were required to pay attention to the sentence with a specific order (e.g., the 2nd sentence being presented). The complexity of our matching paradigm may explain why the best‐performing subject in Experiment I‐a with alternating attention (Fig. 4B) showed decreased performance in experiment II, using the matching paradigm (Fig. 6C).

In addition, the HRTFs used in creating the virtual acoustic space were recorded by other researchers with their experimental settings. It has been shown that head tracking, individualized HRTFs, and room reverberation can improve the externalization quality as well as the localization accuracy (Begault et al. 2001). The virtual acoustic space created in our study was not ideal but sufficient for the subjects to carry out the tasks. More realistic implementations may be considered in the future to achieve better effects.

### Auditory steady‐state response and modulation phase

It is long known that auditory steady‐state response can be elicited by amplitude‐modulated sound (e.g., Picton et al. 1987, 2003; Alegre et al. 2008; Muller et al. 2009). The synchronization to the modulation frequency is highest around 30 to 50 Hz (Galambos et al. 1981). Here, we adopted the “phase‐tagging” algorithm from Lopez‐Gordo and Pelayo (2013) and Lopez‐Gordo et al. (2015) for sound presentation and auditory‐attention decoding. This approach requires that the same amplitude‐modulation frequency is used for all sound tokens, whereas a unique modulation phase is assigned to each spatial position or side (i.e., left/right). The nature of the technique determines that a low modulation frequency must be used to provide enough phase difference (in time).

In general, we found that the modulation phase of a single sound (e.g., control) can be reliably reflected over most of the electrodes. A decision variable based on 90 sec of EEG responses can reach a 100% decoding accuracy with certain electrode channels. The electrodes around C3 and C4 frequently showed low performance. The frontal electrodes also tended to show performance lower than other places, especially when speech was used as the carrier (Fig. 6). In summary, the phase of a single amplitude‐modulated sound with low modulation frequencies (5 or 7 Hz) can be faithfully encoded by EEG response over the majority of electrodes. However, it should be noted that combining multiple trials to form a 90‐sec decision may not be the same as recording continuously over 90 sec.

We would like to point out that the decoding performance truly depended on phase, not other EEG signal features related to spatial attention, such as event‐related potentials. This is because we only extracted the 5 or 7 Hz component of the EEG signal and discarded other frequency components in the analysis. The decision was made according to where this single‐frequency response was located in the complex plane.

### Decoding attention with competing sources required significant averaging

For each attention condition, a total of 800 trials were obtained from each subjects through multiple recording sessions that were not performed on the same day. The combined correlation map (the large heat map on the upper right corner for each plot) indicates the overall performance of individual channels. Anything that was noise would have been washed out after 800 trials. We used this combined plot to choose the “good‐performance electrode” with which a decision will be made for BCI applications. Previous attention studies have featured electrode positions at Cz, Oz, T7, and T8 (Ross et al. 2000; Kim et al. 2011; Lopez‐Gordo and Pelayo 2013). In the present study, the selected electrode position varied for each subject.

When examining the brain patterns obtained from individual sessions using a total duration of 90 sec, there can be significant variations over sessions for some subjects. Those subjects had only five or six sessions out of eight that were significantly above chance. It is likely that some subjects naturally need averaging from more trials to establish a stable performance than others. Some studies on the decoding of auditory attention from multiple sources show that single‐trial responses with a duration of up to 20 sec can yield significant performance (Kim et al. 2011; Lopez‐Gordo et al. 2015). The study by Choi et al. (2013) showed that as short as 3 s of evoked potentials can be used to achieve an accuracy of 65 to 70%. Studies that reconstruct speech envelopes in a cocktail‐party setting require at least 60 sec of EEG responses (O'Sullivan et al. 2015), often combining over multiple trials and many electrodes.

**Figure 7:**
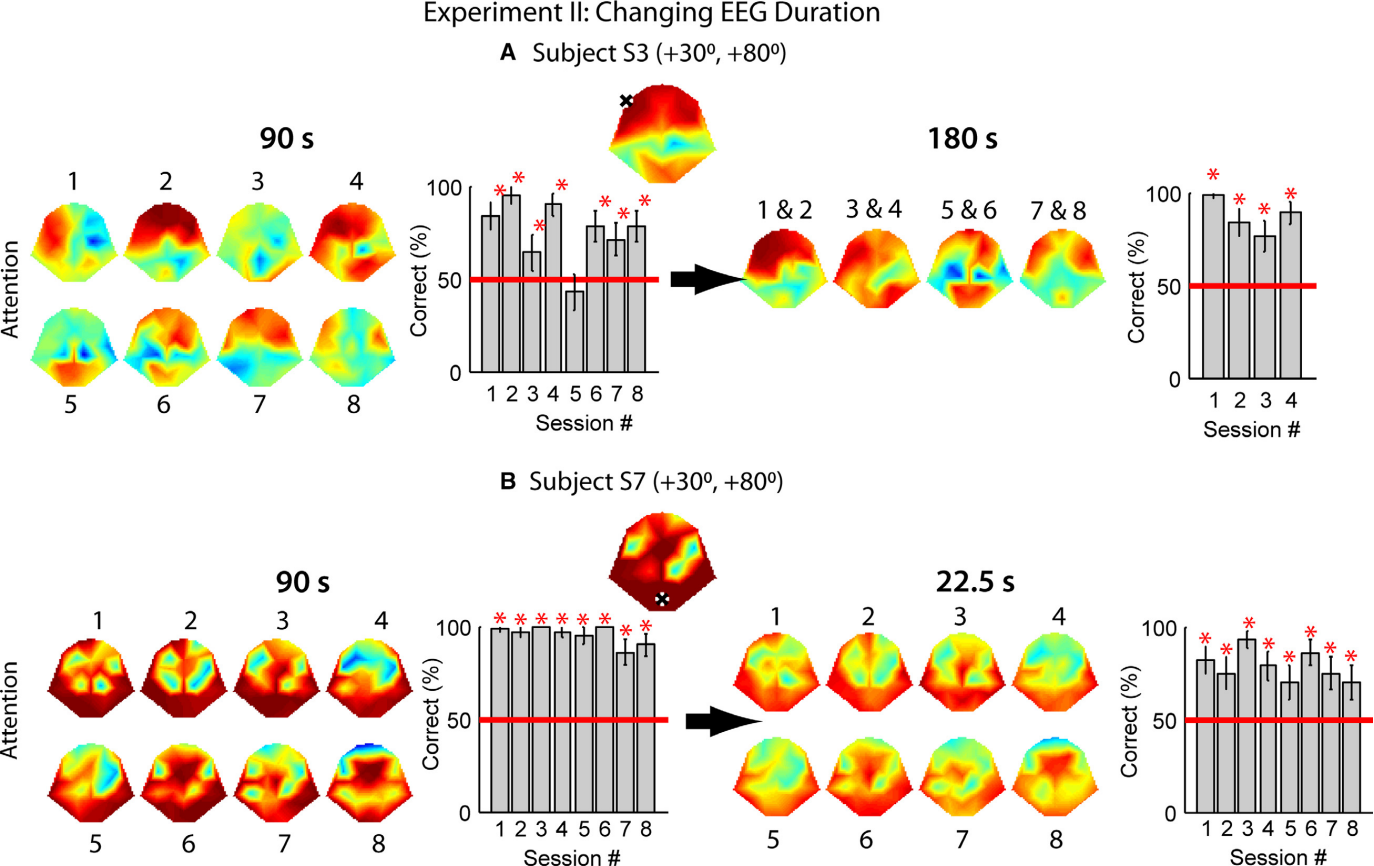
Effects of changing EEG duration. A, decoding performance based on 90‐sec (*left*; re‐plotted from Fig. 6E) or 180‐sec (*right*) EEG responses through a combination of 20 or 40 trials, respectively. Each trial elicited a 4.5‐sec EEG signal. B, decoding performance based on 90‐sec (*left*; re‐plotted from Fig. 6G) or 22.5‐sec (*right*) EEG responses through a combination of 20 or 5 trials, respectively.

Figure 7A is an example of decoding performance based on longer EEG responses. The plot on the left of Figure 7A is replotted from Figure 6E for Subject S3 in Experiment II. The original result was derived from 90‐sec EEG response by combining 20 single trials. If the EEG duration was doubled to 180 sec by averaging over more trials (Fig. 7A, *right*), the performance became better and more stable over time. Each of the small heat maps in Figure 7A (*right*) is a combination of two sessions in Figure 7A (*left*); otherwise there would not have been enough trials in each session for random combinations. Doing so resulted in more stable brain patterns, as well as higher correct rates in the single‐channel performance that varied between 77 and 98% correct (Fig. 7A, *right*). For those subjects, the application of the single‐channel performance would be unsuitable for BCI because of the relatively low efficiency.

In contrast, the best subject performance (Subjects S2 and S7 in Experiment I‐a and Subject S7 in Experiment II) can maintain a high decoding accuracy over all recording sessions using 90‐s EEG response or less, and the brain areas reflecting attention were large. In fact, only 22.5‐sec responses (a combination of 5 trials) for Subject S7 in Experiment II were enough to maintain a performance between 70 and 93% correct (Fig. 7B, *right*). This observation indicates that the phase‐tagging approach has the potential of providing reliable BCI applications with some users but not others.

The variable performance and electrode patterns across subjects are reminiscent of some of the BCI studies based on sensory motor rhythms (Dickhaus et al. 2009). A possible solution is to optimize the decision based on a weighted summation of all electrode channels. O'Sullivan et al. (2015) showed that a regression model with such a weighting algorithm over 128 channels can effectively reconstruct speech envelopes from 60‐sec single‐trial EEG responses. In the present study, detection outcomes from non‐selected electrodes were discarded when applying to a BCI. If a similar weighting approach can be developed for the phase‐tagging algorithm, the performance may be improved for the normal subjects.

In summary, the virtual acoustic space can serve as a portable system to enable decoding of spatial attention for any spatial location. Practically, spatial attention can be decoded in a series of paired sound presentations with decreased angular separations around the target. The phase‐tagging approach may be suitable for some subjects, whereas other subjects may need to explore different sound paradigms such as frequency tagging.

## Conflict of Interest

None declared.
